# Cardiac Computed Tomography Radiomics for the Non-Invasive Assessment of Coronary Inflammation

**DOI:** 10.3390/cells10040879

**Published:** 2021-04-12

**Authors:** Kevin Cheng, Andrew Lin, Jeremy Yuvaraj, Stephen J. Nicholls, Dennis T.L. Wong

**Affiliations:** 1 Monash Cardiovascular Research Centre, Victorian Heart Institute, Monash University and MonashHeart, Monash Health, Clayton, VIC 3168, Australia; kevin.cheng@monash.edu (K.C.); Andrew.lin3@cshs.org (A.L.); jeremy.yuvaraj@monash.edu (J.Y.); stephen.nicholls@monash.edu (S.J.N.); 2 Department of Medicine, Monash University, Clayton, VIC 3168, Australia; 3 Biomedical Imaging Research Institute, Cedars-Sinai Medical Centre, Los Angeles, CA 90048, USA

**Keywords:** machine learning, radiomics, coronary computed tomography angiography, acute coronary syndrome, atherosclerosis, plaque, peri-coronary adipose tissue

## Abstract

Radiomics, via the extraction of quantitative information from conventional radiologic images, can identify imperceptible imaging biomarkers that can advance the characterization of coronary plaques and the surrounding adipose tissue. Such an approach can unravel the underlying pathophysiology of atherosclerosis which has the potential to aid diagnostic, prognostic and, therapeutic decision making. Several studies have demonstrated that radiomic analysis can characterize coronary atherosclerotic plaques with a level of accuracy comparable, if not superior, to current conventional qualitative and quantitative image analysis. While there are many milestones still to be reached before radiomics can be integrated into current clinical practice, such techniques hold great promise for improving the imaging phenotyping of coronary artery disease.

## 1. Introduction

Coronary artery disease (CAD) remains a leading cause of death despite advances in primary and secondary prevention strategies [1]. Acute coronary syndrome (ACS) comprising myocardial infarction (MI) and unstable angina is responsible for much of its mortality and morbidity burden. Vascular inflammation is considered a key driver of atherosclerotic plaque formation and destabilization resulting in ACS [2]. Randomized studies demonstrate a residual inflammatory risk even after the aggressive lowering of low-density lipoprotein cholesterol [3]. The landmark CANTOS trial showed that targeting interleukin-1β with the monoclonal antibody canakinumab reduced recurrent cardiovascular event rates, hence validating the inflammatory hypothesis of atherosclerosis [4]. This has led to a burgeoning research interest in the non-invasive detection of vascular inflammation, which has important implications for cardiovascular risk stratification and the initiation of appropriate risk reduction strategies.

Conventional tests that rely on circulating inflammatory biomarkers (e.g., high-sensitivity C-reactive protein and pro-inflammatory cytokines) are not directly related to the process of atherogenesis, and not specific enough to identify coronary inflammation [5]. Advanced imaging tests such as sodium-fluoride positron emission tomography are costly and not widely available, limiting their applicability in clinical practice [6].

Coronary computed tomography angiography (CCTA) is rapidly becoming a first-line diagnostic test in the investigation of suspected CAD [7,8,9]. It offers a unique non-invasive modality to image and assess the coronary lumen, plaque, and perivascular tissue [10]. Currently, CCTA interpretation predominantly relies on visual assessment which disregards the large volume of three-dimensional datasets recording each pixel’s radiodensity and their relationship to each other. Recently, improvements with CCTA’s imaging quality and access to high-performance computing have led to the development of cardiac computed tomography radiomics, introducing the field of artificial intelligence (AI) to cardiovascular imaging. Radiomics is the process of extracting numerous quantitative features from a given region of interest to create large data sets in which each abnormality is described by hundreds of parameters. Data mining is the process of finding new, meaningful patterns and relationships between the different variables. Machine learning can also be applied to the analysis of radiomics parameters. From these results, novel imaging biomarkers may be identified that can increase the diagnostic accuracy of CCTA and expand our knowledge of the underlying pathologic processes.

The objective of this review is to first introduce the current state of AI in modern CCTA, followed by an overview of the coronary radiomics analysis workflow, current literature regarding the use of radiomics techniques in the assessment of coronary inflammation, and finally the current challenges of incorporating radiomics into clinical practice.

## 2. Artificial Intelligence, Machine Learning and Big Data

Modern cardiovascular medicine generates a vast amount of biomedical, imaging, and clinical data as part of patient care delivery. The high dimensionality of data poses demanding analytical challenges but offers rich opportunities for improved discovery. The term *big data* refers to extremely large datasets which cannot be analyzed, interpreted, or stored efficiently using conventional data-processing techniques [11]. In healthcare, this includes “omic” data (genomics, proteomics, or lipidomics), biometrics from streaming mobile devices, clinical data from electronic health records, and image-derived information.

Traditional statistical methods cannot efficiently handle and learn from such elaborate data sets to develop diagnostic and predictive models for assisting clinical decision-making. *Artificial intelligence* refers to the use of computational techniques to perform tasks that are characteristic of human intelligence, such as visual perception, pattern recognition, planning, problem-solving, and decision making [12]. AI is being increasingly applied in cardiovascular imaging for image segmentation, automated measurements, and risk stratification [13,14].

*Machine learning* (ML) is a subset of AI which uses computer algorithms with the ability to automatically learn to perform a task and improve from experience by being exposed to a large amount of data, without being explicitly programmed in decision making [15]. ML methods complement and extend existing statistical methods, providing tools and algorithms to understand patterns from large, complex, and heterogeneous data. Although conventional statistical methods are capable of both discovery and prediction, ML methods are suitable and generalizable across a variety of data types and offer analyses and interpretation across complex variables [16]. Additionally, ML techniques typically rely on fewer assumptions and provide superior and more robust predictions.

Common types of ML algorithms include supervised and unsupervised [17]. The selection of the right model often relies on the operator’s expertise, the nature of the dataset, and the purpose of the final AI system [18]. *Supervised learning* algorithm is most commonly employed in CCTA application, which uses a labeled dataset to predict the desired outcome. This involves the iterative selection, processing, and weighting of individual features to learn the underlying patterns within the data that best fit the given outcome. Limitations of supervised learning include the need for large, labeled training datasets and validation datasets. It is also often time-consuming due to the need for the manual labeling of large amounts of data. Furthermore, supervised algorithms are limited to predicting known outcomes. In *unsupervised learning*, unlabeled data are used to predict unknown outcomes, and the algorithm must discover inherent patterns within the dataset. Such techniques include principal component analysis and the wide array of clustering algorithms (e.g., “k-means” or hierarchical clustering) which cluster data into groups based on similarity. The major challenge in unsupervised learning is difficulty in identifying the initial cluster pattern (how many clusters there are, and their respective boundaries), which may lead to overfitting of the model to the dataset. Hence, these models require validation in multiple cohorts.

## 3. Radiomics: Bringing CCTA Imaging into the Age of Artificial Intelligence

CCTA is now recognized as the pivotal non-invasive diagnostic imaging modality of choice for cardiovascular risk stratification and the assessment of stable and unstable cardiovascular patients [19,20]. The strength of CCTA lies in its ability to reliably exclude coronary stenosis and [21,22], to directly visualize the vessel wall and plaque morphology [23]. Over the past years, CCTA technology has developed at unprecedented speed while data analysis and image interpretation progressed at a slower pace as its diagnostic potential is burdened by a certain degree of subjective visual assessment and inter-reader variability. For example, even among expert readers, the inter-reader reproducibility of high-risk plaque features is highly variable (κ range 0.15–0.34) [24]. Furthermore, it is unable to detect the finer characteristics of high-risk plaques such as macrophage activity, neovascularization, plaque rupture, and plaque erosion, which can all be detected by optical coherence tomography (OCT) [25].

CT radiodensity of the vascular tissue has been shown to be a good surrogate marker of histological composition as correlated with intravascular ultrasound (IVUS) [26]. Therefore, CCTA’s capability to non-invasively acquire isotropic three-dimensional data creates a unique opportunity to analyze complex spatial image patterns using *radiomics*. This refers to the process of extracting a large number of quantitative imaging features from a given region of interest to create big data in which each abnormality is characterized by hundreds of parameters extending far beyond those that can be characterized by the human eye [27]. The quantitative features are calculated using dedicated software, which accepts the image datasets as inputs. Following feature extraction, data mining and ML approaches are used to find new, meaningful patterns between the different parameters to identify novel imaging biomarkers which may reflect the underlying pathophysiology of a tissue. Additionally, radiomics offers mathematic objectivity with the use of quantitative image parameters instead of qualitative markers to express different lesion characteristics.

The transition from qualitative to quantitative radiomics assessment was initiated by oncoradiology and has proven to be a valuable tool [28]. Several studies have shown radiomics to improve the diagnostic accuracy, staging, and grading of cancer, response assessment to treatment, and predict clinical outcomes [29,30,31,32,33,34,35,36]. CCTA radiomics faces its unique technical challenges as atherosclerotic lesions have a significantly smaller number of voxels for analysis than tumors as well as having complex geometric shapes.

## 4. Overview of Coronary Radiomics Workflow

### 4.1. Step 1: Images Acquisition

The coronary radiomics workflow begins with the CCTA image, which is represented as a three-dimensional dataset of different attenuation values using semi-parametric calibrated Hounsfield Units (HU). Each of the different tissues involved absorbs radiation to a different extent and thus, they are depicted as having different attenuation values on CT. As such, each voxel is a separate measurement of how much radiation is absorbed in the given volume and correlates with the underlying biology.

### 4.2. Step 2: Region-of-Interest Segmentation

**Figure 1:**
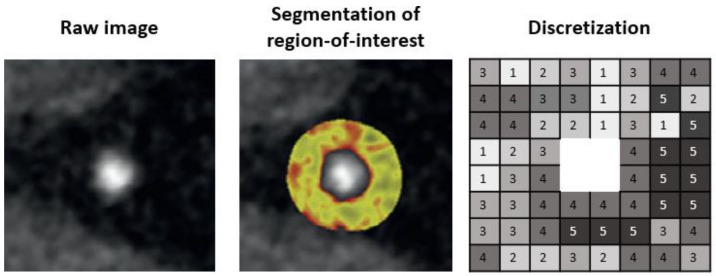
Pipeline for segmentation of region-of-interest.

Next, a region-of-interest (ROI) is defined so that only information related to the lesion can be extracted. The coronary artery needs to be segmented at its proximal and distal ends of interest as well as determining the inner and the outer vessel wall boundaries. Then, the HU values of the voxels need to be discretized into a given number of groups, as voxels with the identical value rarely occur in medical imaging (Figure 1).

To date, segmentation of the coronary artery is done either manually or semi-automatically with dedicated software such as QAngioCT, 3D Slicer, or AutoPlaque [37,38,39]. Automatic lumen and vessel contours can be manually edited if needed. Software such as 3D Slicer has incorporated an installable plugin for the open-source PyRadiomics package for integrated radiomics analyses. Manual and semi-automated image segmentation can be time-consuming and prone to observer bias and variability. Therefore, studies using such segmentation technique should assess for intra- and inter-observer reproducibility of the derived radiomic features and exclude non-reproducible features from further analyses. Deep learning-based image segmentation is yet to be available for coronary assessment but it is rapidly emerging for many different organs [40]. This has the advantage of avoiding intra- and inter-observer variability of radiomic features. However, the generalizability of trained algorithms is a major limitation currently, and applying those algorithms on a different dataset often results in complete failure. Thus, further research is needed for the development of robust and generalizable algorithms for automated image segmentation.

### 4.3. Step 3: Radiomic Features Extraction

Feature extraction refers to the calculation of feature descriptors to quantify the characteristics of the grey levels within the ROI. Since many different ways and formulas exist to calculate those features, adherence to the Image Biomarker Standardization Initiative guideline is recommended [41]. These radiomic features can be broadly grouped into four major categories: (1) shape based, (2) intensity based, (3) texture based, and (4) transformed based (Table 1). In practice, feature extraction means simply running the dedicated software package and waiting for the computation to be finished.

*Shape*-*based metrics* are more widely used in clinical routines and easily comprehensible. It describes the shape of the traced ROI and its geometric properties such as volume and the maximum diameter along different orthogonal planes.

*Intensity*-*based metrics* describe the distribution of individual voxel values but without accounting for their spatial relationships. These are histogram-based properties reporting the average and variation (mean, median, maximum, and minimum values of the voxel intensities on the image). The shape of the distribution can be quantified by skewness (asymmetry) and kurtosis (flatness). Lastly, the heterogeneity of the sample values can be quantified by uniformity and randomness (entropy).

**Figure 2:**
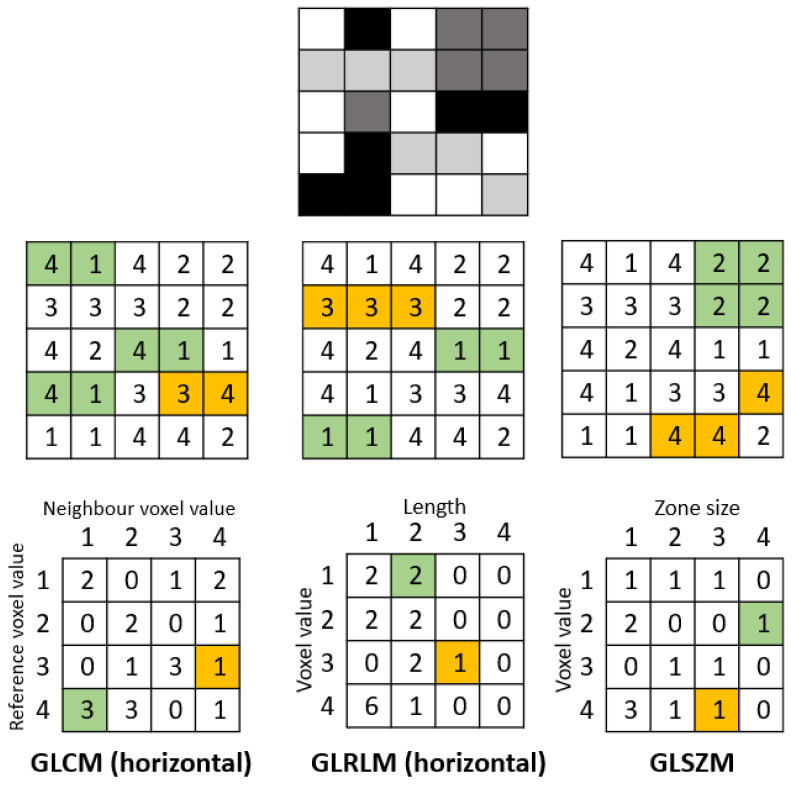
Example calculation of radiomic texture features. Whereas the gray-level co-occurrence matrix (GLCM) relies on pixel pairs, the gray-level run-length matrix (GLRLM) relies on runs, and the gray-level size zone matrix (GLSZM) relies on areas of neighboring pixels with the same gray-level.

*Texture*-*based metrics* are obtained by calculating the statistical inter-relationship between neighboring voxels [42]. They provide a measure of the spatial arrangement of the voxel intensities, and hence of intra-lesion heterogeneity. The gray-level co-occurrence matrix (GLCM) is a matrix whose row and column numbers represent gray values, and the cells contain the number of times corresponding gray values are in a certain relationship (angle, distance), as shown in Figure 2. Features calculated on GLCM include entropy (related to heterogeneity), energy (also defined as angular second moment, also describes heterogeneity of an image), contrast (measures local variation), cluster shade (sensitive to heterogeneity), homogeneity, dissimilarity, and correlation [43].

Gray-level run-length matrix (GLRLM) quantifies consecutive voxels with the same intensity along fixed directions (Figure 2) [44]. It is represented as a two-dimensional matrix in which each element describes the number of times a gray level appears consecutively in the direction specified. Gray-level size zone matrix (GLSZM) is a matrix in which the elements at row r and column s store the number of zones (the connected voxels with the same gray level) with gray level r and size s (Figure 2).

An image is represented in the spatial domain where a vast number of pixel/voxel values are distributed along with the spatial coordinates. The image can be *transformed* into the frequency domain by representing the pattern and rate at which the image intensity values change along with spatial directions. One method is the wavelet transformation which decomposes the data into high- and low-frequency components. At high frequency, the wavelets can capture discontinuities, ruptures, and singularities in the original data. At low frequency, the wavelets characterize the coarse structure of the data to identify the long-term trends. Thus, numerous hidden and significant temporal features of the original data can be extracted while improving the signal-to-noise ratio of imaging studies [45].

### 4.4. Step 4: Feature Selection/Dimensionality Reduction

The process of feature extraction will yield a large number of radiomics features. Using all the extracted features in a statistical model would lead to overfitting, where the model corresponds too closely to the training dataset, such that it picks up noise and performs poorly in internal and external validation. The purpose of feature selection is to identify the optimal set of radiomics features to be taken forward for model building and aim to include model features that are most informative and robust while removing those that are unstable or provide repetitive information.

**Figure 3:**
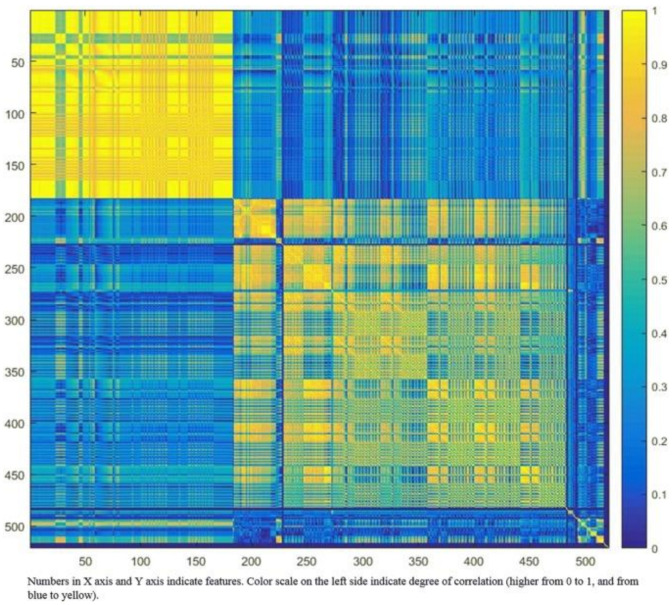
Graphic representation of radiomic feature clustering. Each radiomic feature was compared with all other features using linear regression analysis. Features were clustered based on the absolute values of the correlation coefficient of the corresponding regression models and plotted along both axes (ranging from 0 to 1 with greater values are shown in yellow with increasing intensity). In this example, the yellow blocks along the diagonal identify the clusters containing the highly correlated radiomic features. The first cluster in the top left corner demonstrated very high redundancy for radiomic features (represented by the high homogeneity of the yellow blocks). The blue blocks visualize the low correlation observed between the radiomic features. Adapted from Rizzo et al. [47].

Robustness of features can be assessed through test–retest, with the removal of those with poor repeatability. Many radiomics features will be expected to reflect duplicate information (for example, the diameter, surface area, and volume of a sphere shape), and these will need to be identified and to select only those that are most informative. Cluster analysis aims to create groups of similar features (clusters) with high intra-cluster redundancy and low inter-cluster correlation. This is often depicted by a cluster heat map as shown in Figure 3. A single feature may be selected from each cluster as representative and used in the following association analysis. Principal component analysis through different methods reduces the extracted features to a subset that provides nearly as much information as the whole feature set [46].

### 4.5. Step 5: Model Building

Once the final sets of radiomics features are identified, they can be used as predictor/discriminatory variables of the classification model. The model building starts by using a training set consists of example cases (training examples) with input vectors consists of the final set of radiomic features which are paired with desired model output labeled with the known outcome. The algorithm determines how much weight (importance) is placed on each feature to achieve optimal model performance. In some cases, logistic regression will be adequate to address a simple classification problem. More commonly, machine learning algorithms (random forest, neural networks, or least absolute shrinkage and selection operator) are used to train different models; from these, the model with the best performance is selected.

### 4.6. Step 6: Validation

Before the predictive model can be applied in a clinical setting, the model’s stability and reproducibility must be assessed. The first step in model validation is *internal cross*-*validation* which uses an internal dataset that has not mixed with the training data during the model building or feature selection process.

*External validation* with an independent external dataset is important for the assessment of model performance and generalizability. The models are able to compute a probability of belonging to a class and not only a discrete value. Model performance is thus assessed using measures of sensitivity, specificity, receiver operating curves, and area under the curve (AUC).

## 5. Current Literature on Radiomics Analysis for Coronary Artery Disease

Recently, there has been increasing research interest in CCTA radiomics to identify new biomarkers associated with plaque vulnerability (Table 2). This has been facilitated by the ever-growing size of CCTA datasets and registries, and its unique capability to capture reliable qualitative and quantitative information of coronary plaques and the surrounding adipose tissue on the entire coronary tree.

### 5.1. Radiomics Assessment of Coronary Plaques

Coronary atherosclerotic plaques consist of distinct histological components with different attenuation values on CTA; each voxel is a separate measurement of the amount of radiation absolved in the given volume. This has enabled the assessment of plaque morphology in vivo, and several qualitative and quantitative imaging biomarkers are known to associate with adverse cardiovascular events. Advanced atherosclerotic plaques that are prone to cause ACS are characterized by large lipid-rich necrotic cores, increased amounts of inflammatory cells, and thin fibrous caps [48]. Invasive imaging modalities, such as IVUS and OCT offer sub-millimeter spatial resolution and can depict distinct morphologic markers of plaque vulnerability, which have been validated by histology and clinical investigations [49,50]. While CCTA might not have sufficient spatial resolution, its unique ability to non-invasive image atherosclerotic lesions holds great potential to identify high-risk plaques. Four distinct plaque characteristics (positive remodeling, low attenuation, spotty calcification, and napkin-ring sign) derived from CCTA have been linked to major adverse cardiovascular events [10]. However, these visually detectable adverse plaque characteristics show only a modest correlation with IVUS- or OCT-derived features [51].

**Table 2:** Studies examining the relationship between CT-derived radiomics parameters of coronary plaques and PCAT with coronary atherosclerosis.

Study	Input Region-of-Interest for Radiomics Analysis	Outcomes Assessed	Study Design	Main Findings
Kolossváry et al. [37] (2017)	Coronary artery plaques	Napkin-ring sign	30 plaques with napkin-ring sign vs. 30 matched plaques without.	Best radiomic parameter: short-run low-gray-level emphasis (AUC 0.92, CI 0.82–0.996). Best conventional parameter: mean plaque attenuation (AUC 0.77, CI 0.64–0.88).
Kolossváry et al. [52] (2019)	Coronary artery plaques	Advanced atherosclerotic lesions	445 histologic cross-sections: 311 early atherosclerotic lesions (adaptive intimal thickening, pathologic intimal thickening, and fibrous plaque). 134 advanced atherosclerotic lesions (early fibroatheroma, late fibroatheroma, and thin-cap fibroatheroma). Lease angles regression machine learning model used.	Radiomics-based machine learning (AUC 0.73 CI 0.63–0.84). Visual-based plaque attenuation pattern (AUC 0.65, CI 0.56–0.73). Histogram-based low attenuation plaque area (AUC 0.55, CI 0.42–0.68). Histogram-based average HU (AUC 0.53, CI 0.42–0.65).
Kolossváry et al. [53] (2019)	Coronary artery plaques	IVUS attenuated plaque OCT thin-cap fibroatheroma NaF^18^-PET positivity	25 patients (44 lesions) undergoing CCTA, NaF^18^-PET, IVUS, and OCT.	*IVUS attenuated plaque* Best radiomic parameter: fractal box counting dimension of high attenuation voxels (AUC 0.72, CI 0.65–0.78) Best conventional parameter: non-calcified plaque volume (AUC 0.59, CI 0.57–0.62) *OCT thin-cap fibroatheroma* Best radiomic parameter: fractal box counting dimension of high attenuation voxels (AUC 0.80, CI 0.72–0.88) Best conventional parameter: presence of low attenuation (AUC 0.66, CI 0.58–0.73) *NaF^18^-PET positivity* Best radiomic parameter: surface of high attenuation voxels (AUC 0.87, CI 0.82–0.91) Best conventional parameter: presence of two high risk features (AUC 0.65, CI 0.64–0.66)
Oikonomou et al. [38] (2019)	PCAT of proximal RCA and proximal LAD coronary artery.	*Study 1* TNFA expression (inflammation) COL1A1 expression (fibrosis) CD31 expression (vascularity) *Study 2* Major adverse cardiovascular events (composite endpoint of cardiac mortality and non-fatal myocardial infarction) *Study 3* Acute myocardial infarction vs. stable CAD	*Study 1* 167 patients underwent CCTA followed by cardiac surgery where cardiac adipose tissues were obtained. *Study 2* 5487 patients underwent CCTA as part of the SCOT-HEART trial or the CRISP-CT study. Out of this cohort, 101 patients experienced MACE within 5 years of CCTA. Random forest machine learning model used. *Study 3* 44 patients with AMI vs. 44 matched controls with stable CAD.	*Study 1* TNFA expression is best associated with wavelet-transformed mean attenuation. COL1A1 and CD31 expression best associated with texture-based metrics. *Study 2* Radiomics-based ML model significantly improved MACE prediction when added to traditional risk stratification that included risk factors, coronary calcium score, coronary stenosis, and CCTA HRP features (Δ[C-statistic] = 0.126, *p* < 0.001). *Study 3* Fat radiomics profile is higher in patients with AMI compared to the matched controls (*p* < 0.001).
Lin et al. [39] (2020)	PCAT of proximal RCA	Myocardial infarction vs. stable CAD vs. No CAD	60 patients with acute MI were matched with 60 controls. Extreme gradient boosting machine learning model used.	20.3% of the radiomic parameters differed significantly between MI patients and controls. 16.5% differed between patients with MI vs. stable CAD. No difference between patients with stable CAD vs. control.
Kolossváry et al. [54] (2021)	Coronary artery plaques	Elevated CVD risk (ASCVD score ≥ 7.5%) HIV infection Cocaine use	300 patients with subclinical CAD who had serial CCTA at least 1 year apart. 168 (56%) had an increased ASCVD score. 226 (75.3%) had HIV infection. 174 (58%) reported cocaine use.	Elevated ASCVD score was associated with 8.2% of radiomic features, HIV infection was associated with 1.3% and cocaine use was associated with 23.7%. Parameters associated with elevated ASCVD score or cocaine use and HIV infection did not overlap.

AMI: acute myocardial infarction, ASCVD: atherosclerotic cardiovascular disease, AUC: area under the curve, CAD: coronary artery disease, CCTA: coronary computed tomography angiography, CD31: cluster of differentiation 31, CI: confidence interval, COL1A1: collagen type 1 alpha 1, CVD: cardiovascular disease, HIV: human immunodeficiency virus, HRP: high-risk plaque, HU: Hounsfield unit, IVUS: intravascular ultrasound, LAD: left anterior descending, MACE: major adverse cardiovascular events, MI: myocardial infarction, ML: machine learning, NaF^18^-PET: sodium fluoride-18 positron emission tomography, OCT: optical coherence tomography, PCAT: peri-coronary adipose tissue, RCA: right coronary artery, TNFA: tumor necrosis factor alpha.

The first study to perform radiomics analysis of the coronary artery demonstrated the feasibility and potential clinical utility of CCTA radiomics to reliably identify plaques with the napkin-ring sign [37]. This high-risk plaque phenotype is composed of a thin fibrotic cap above a lipid-rich necrotic core, an extracellular conglomerate within the intima induced by necrosis and apoptosis of lipid-laden macrophage foam cells [55]. This qualitative CT feature is defined as a plaque cross-section with a central area of low CT attenuation in contact with the lumen, which is surrounded by a ring-shaped higher attenuation tissue. Kolossváry et al. compared 30 patients with plaques with the napkin-ring sign to 30 matched patients with plaques without such sign but with similar degrees of calcification, luminal obstruction, localization, and acquisition parameters. The study showed that 20.6% of radiomic features showed significantly higher discrimination of the napkin-ring sign than conventional quantitative measures. Furthermore, parameters incorporating the spatial distribution of voxels (GLCM, GLRLM, and geometry-based parameters) have a better predictive value than first-order statistics.

In a subsequent study, the radiomics-based ML model has been shown to outperform conventional and histogram-based CCTA analysis in differentiating between early and advanced atherosclerotic lesions identified by histologic cross-sections [52]. Analyzing 21 coronary arteries obtained ex vivo from seven male donors, lesions were considered advanced if early fibroatheroma, late fibroatheroma, or thin-cap atheroma was found. Eight different radiomics-based ML models were tested, and the least angles regression models provided the best discriminatory power on the training set. The radiomics-based ML model outperformed expert visual assessment for the identification of advanced lesions (AUC 0.73 vs. 0.65, *p* = 0.04).

More recently, sodium-fluoride positron emission tomography (NaF^18^-PET) has been introduced as a radionuclide imaging modality to identify inflammation and microcalcifications in coronary atherosclerotic plaques [56]. Vascular calcification is viewed as a cellular response to a necrotic, inflammatory microenvironment and also a marker of plaque metabolic activity [57]. Detecting areas of microcalcification at its earliest stages can help to identify high-risk lesions, but these do not become detectable on CCTA until late in the natural history of atherosclerosis [56,58]. In a retrospective analysis, Kolossváry et al. were able to demonstrate that CCTA radiomic parameters (compared to conventional CT parameters) consistently correlates better with the invasive and radionuclide imaging markers of high-risk plaques [53]. Among the calculated radiomic parameters, textural features (fractal dimensions) correlated best with attenuated plaque identified by IVUS (AUC 0.72, CI 0.65–0.78) and thin-cap fibroatheroma by OCT (AUC 0.80, CI 0.72–0.88). While the surface of high attenuation voxels correlated best with NaF^18^-positivity (AUC 0.87, CI 0.82–0.91). These microcalcifications were most likely recorded as voxels with higher HU values but were visually imperceptible. Furthermore, since the microcalcifications are not grouped in one cluster like the calcified plaques, the surface area of these high attenuation voxels will be larger.

It has been shown that both conventional and nonconventional cardiovascular risk factors (such as cocaine use and HIV infection) affect the different pathways of atherosclerosis progression at a molecular level [59,60,61]. Cocaine use impairs nitric oxide release and increases the levels of cell and leukocyte adhesion molecules, causing the migration of white blood cells into the intimal layers. HIV infection causes the chronic activation of the innate immune system, which results in increased levels of inflammatory cytokines (such as interleukin-6, CD14, and CD163) and activation of white blood cells resulting in chronic vasculopathy. In a study involving 300 patients with subclinical CAD, Kolossváry et al. demonstrated that cocaine use, HIV infection and elevated atherosclerotic cardiovascular disease risk were each associated with their own distinct sets of radiomics parameters (23.7%, 1.3%, and 8.2%, respectively) [54].

### 5.2. Radiomics Assessment of Pericoronary Adipose Tissue

Beyond plaque, it is now established that the coronary artery wall and its pericoronary adipose tissue (PCAT) interact in a bidirectional manner [58]. Exposure of PCAT to pro-inflammatory cytokines suppress the differentiation of pre-adipocytes while triggering their proliferation, resulting in numerous smaller adipocytes with fewer intracellular lipid droplets [58]. This creates a gradient of differing PCAT density with a lipid-rich/less-aqueous phase adjacent to a non-diseased vessel to lipid-poor/more-aqueous phase adjacent to an inflamed artery. This inflammatory process was paralleled by reduced gene expression of the adipocyte differentiation markers PPARγ, CCTAT/enhancer binding protein α (CEBPA), and fatty acid binding protein-4 (FABP4).

Routine CT employs a Hounsfield Units scale of attenuation (reduction in signal), which can be used as a non-invasive measure of adipose tissue (AT) quality [62]. AT is detected within the window of −190 to −30 HU [63,64], and experimental animal studies have shown lower HU to be associated with more lipid-dense AT [65]. The link between biopsy-proven PCAT inflammation and CT attenuation was established in a landmark study by Antonopoulos et al. [58]. On ex vivo CT scans of AT explants and in vivo CCTA, they demonstrated an inverse association of PCAT attenuation with histological adipocyte size and degree of adipocyte differentiation, with higher PCAT attenuation (less negative HU) reflecting smaller adipocytes with lower lipid content. This surrogate measure of coronary inflammation has been shown to predict plaque progression and cardiac mortality in patients undergoing CCTA for suspected CAD and to differentiate stages of CAD [15,57,66,67]. However, PCAT attenuation is akin to an intensity-based parameter that simply enumerates the average voxel intensity values, without considering the spatial relationship among voxels.

Following on from their landmark study establishing the utility of PCAT attenuation, Oikonomou et al. performed radiomics analysis of their original cohort of 167 patients [38]. The authors found that wavelet-transformed mean attenuation (an intensity-based metric) was most correlated with the relative expression of TNF-α (a surrogate marker of inflammation). Higher-order features (such as small area low gray-level emphasis, short-run low gray-level emphasis, and informal measure of correlation) correlated with relative expression of COL1A1 (a surrogate marker of fibrosis) and CD31 (a surrogate marker of vascularity). The authors subsequently developed a ML model (random forest) using a pool of 5487 patients who underwent CCTA from either the CRISP-CT study or the SCOT-HEART trial [15,68]. The selected CCTAs were then randomly split into a training/internal validation (80%) and an external validation set (20%). In total, 1391 radiomic features for PCAT were included in the model, from 101 patients who presented with MACE at 5 years, against 101 matched controls. Compared with established clinical risk prediction models, the used algorithm was able to accurately discriminate cases over controls both in the validation study and when applied to the SCOT-HEART study population (∆C-statistic = 0.126, *p* < 0.001). In the third part of their study, the authors have demonstrated that there is a significant difference in the radiomics profile between their cohort of 44 patients with AMI compared with 44 matched controls. Interestingly, in a subset of 16 patients who underwent repeat CCTA 6 months later, there was no significant change with the radiomics profile but there was a significant decrease with the Fat Attenuation Index (intensity-based metrics).

On the other hand, radiomics analysis of PCAT performed by Lin et al. showed that textural (GLCM and GLRLM) and geometric, rather than intensity-based radiomic features, to be most significant in distinguishing patients with and without MI [39]. Consistent with the previous study, the authors found no significant change in the radiomics profile at 6 months of follow-up. This study utilized a state-of-the-art boosted ensemble ML algorithm (XGBoost) to create a predictive model for identifying patients with MI [69]. Radiomic features provided incremental value over and above PCAT attenuation and clinical features (including hs-CRP and cardiac risk factors) for discriminating patients with MI.

## 6. Challenges and Future Perspectives

Radiomics is an exciting new discipline with the potential to increase our knowledge in CCTA imaging inform downstream decision making. However, like most emerging techniques, there is a need for standardized acquisition protocols and data analysis techniques to provide a robust framework for radiomics analysis. Several studies have shown that imaging parameters, reconstruction settings, or segmentation algorithms all affect the radiomics signature of lesions (Table 3) [70,71,72,73]. Furthermore, it has been shown that the variability caused by these changeable parameters is in the range or even greater than the variability of radiomic features of tumor lesions [74]. To date, there are several contributions that aim to facilitate standardization of radiomics implementation, and data reporting [41,75,76].

Additionally, the current radiomics analysis workflow remains technically complex and time-consuming to be a useful addition to the daily clinical routine. There is a need to develop user-friendly automated software solutions capable of implementing radiomics without increasing the clinical load.

In the future workflow, a useful radiomics tool should seamlessly integrate into the clinical radiological workflow and be incorporated into or interfaced with the existing RIS/PACS system. Such system should provide a deep learning-based fully automated segmentation tool with the option for manual correction. Known important radiomics features could then be displayed alongside other quantitative imaging biomarkers and the images themselves. The clinician could then use all the available information to precisely predict the patient’s cardiovascular risk and prescribed personalized preventive therapy.

Lastly, CCTA radiomics is an emerging research tool and there is a need for prospective data, multi-center studies, and cost analysis before it has the potential for implementation into clinical workflow. It is also vital to have large datasets available to optimize external validation and enhance the generalizability of the prediction models. This can be facilitated by cooperation between academic institutions.

## 7. Conclusions

For many years, CCTA was regarded as a rule-out test for obstructive coronary artery disease due to its excellent negative predictive value. Radiomics presents a novel quantitative image analysis technique with the potential to greatly augment CCTA phenotyping in a manner that enhances our diagnostic and predictive capabilities. CCTA radiomics features may also provide unique insights into the pathophysiology of atherosclerosis at the tissue level and aid understanding of the mechanism of the “vulnerable plaque”. However, current clinical data remains in its infancy and further effort is needed to standardize radiomics analysis protocols among different centers. Moreover, several technical aspects need to be further investigated to ensure the reliability and generalizability of the radiomic features. Furthermore, before radiomics can become of the daily clinical routine, further effort is required to render such technology sufficiently user friendly and time effective. Despite its present technical challenges, there is great promise for radiomics to facilitate an individualized assessment of cardiovascular biology and risk.

## Figures and Tables

**Table 1:** Classification of different radiomic features.

Classification	Metrics	
Shape (geometry)	1-dimensional (major axis, minor axis) 2-dimensional (surface area) 3-dimensional (volume, sphericity, spherical disproportion, compactness, elongation, flatness) Fractal dimension	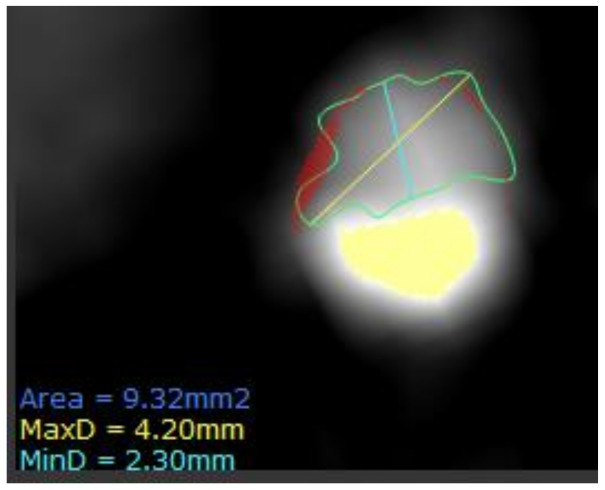
Intensity	Average and spread Shape Diversity	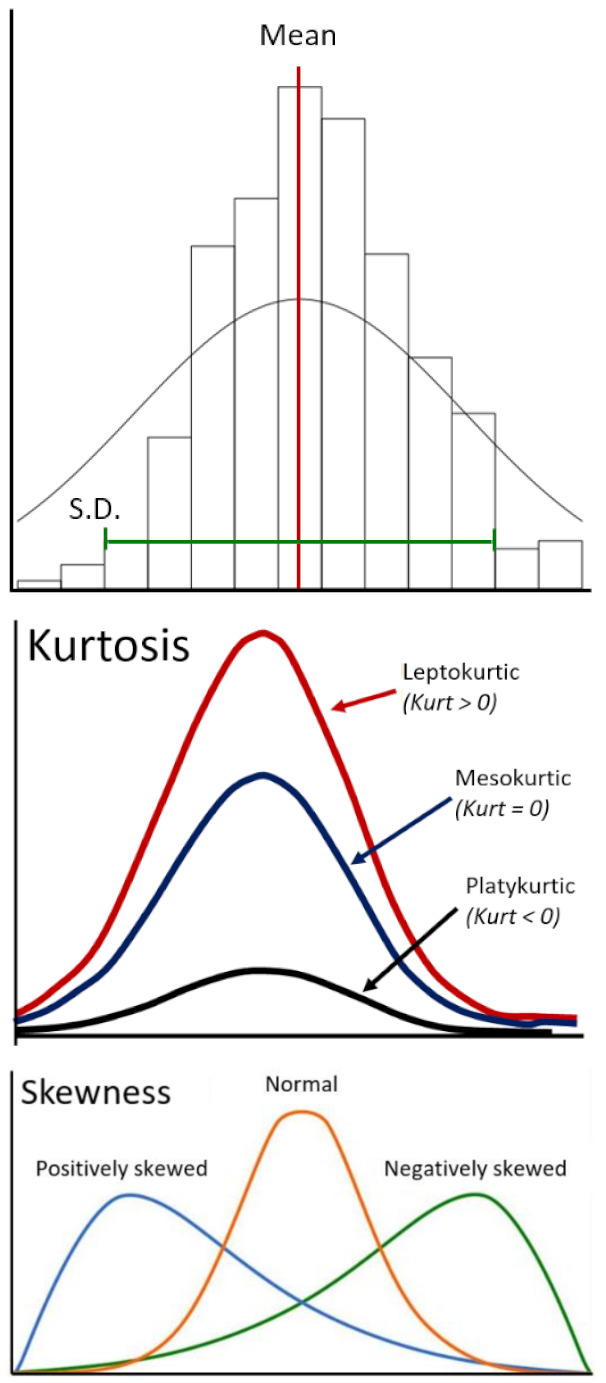
Texture	Gray-Level Co-occurrence Matrix (GLCM) Gray-Level Run-length Matrix (GLRM) Gray-Level Size Zone Matrix (GLSZM) Neighborhood Gray-Tone Difference Matrix (NGTDM)	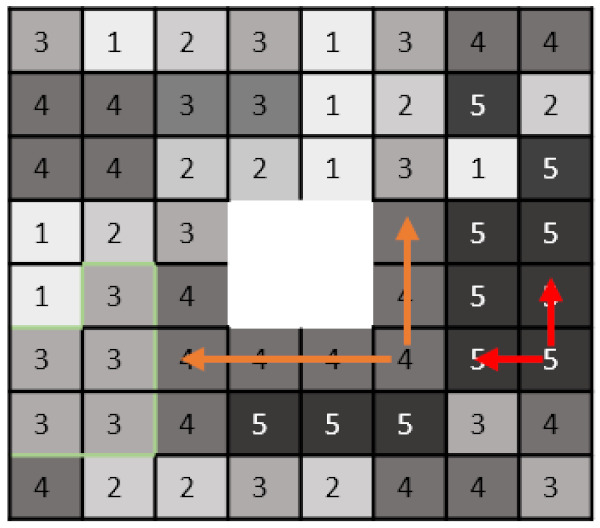
Transform	Fourier transform Gabor transform Wavelet transform	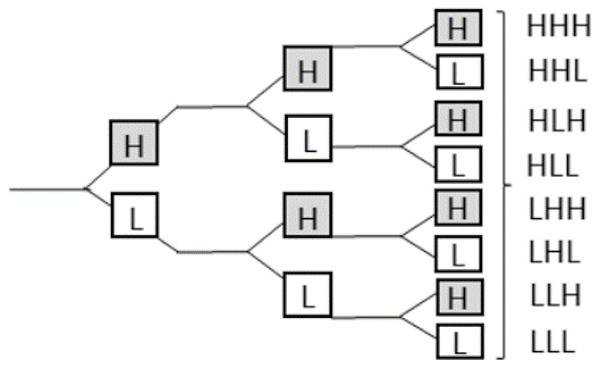

**Table 3:** Potential factors limiting radiomic feature robustness, reproducibility, and classification performance.

Image Acquisition	Reconstruction	Segmentation and Post-Processing	Feature Extraction	Model Building and Validation
Tube voltage and milliamperage Slice thickness Field of view/pixel spacing Acquisition mode Contrast timing Vendor	Reconstruction matrix Slice thickness Reconstruction kernel Reconstruction technique	Manual operator technique Semi-automated algorithm Size of the ROI HU discretization	Number and types of radiomics parameters Extraction algorithm	Algorithm selection Population of the validation sets

HU: Hounsfield unit, ROI: region of interest.
